# The chloroplast protein HCF164 is predicted to be associated with *Coffea* S_H_9 resistance factor against *Hemileia vastatrix*

**DOI:** 10.1038/s41598-023-41950-4

**Published:** 2023-09-25

**Authors:** Leonor Guerra-Guimarães, Carla Pinheiro, Ana Sofia F. Oliveira, Andrea Mira-Jover, Javier Valverde, Fernanda A. de F. Guedes, Herlander Azevedo, Vitor Várzea, Antonio Jesús Muñoz Pajares

**Affiliations:** 1 CIFC - Centro de Investigação das Ferrugens do Cafeeiro, Instituto Superior de Agronomia, Universidade de Lisboa, Tapada da Ajuda, 1349-017 Lisboa, Portugal; 2 LEAF - Linking Landscape, Environment, Agriculture and Food Research Center, Associated Laboratory TERRA, Instituto Superior de Agronomia, Universidade de Lisboa, Tapada da Ajuda, 1349-017 Lisboa, Portugal; 3 UCIBIO Applied Molecular Biosciences Unit, Department of Life Sciences, NOVA School of Science and Technology, Universidade NOVA de Lisboa, 2829-516 Caparica, Portugal; 4 Associate Laboratory i4HB Institute for Health and Bioeconomy, NOVA School of Science and Technology, Universidade NOVA de Lisboa, 2829-516 Caparica, Portugal; 5 Center for Computational Chemistry, School of Chemistry, University of Bristol, University Walk, Bristol, BS8 1TS UK; 6 Departamento de Genética, Universidad de Granada, 18071 Granada, Spain; 7 Área de Ecología, Departamento de Biología Aplicada, Universidad Miguel Hernández, Elche, Spain; 8 CIBIO, Centro de Investigação em Biodiversidade e Recursos Genéticos, InBIO Laboratório Associado, Universidade do Porto, Campus de Vairão, 4485-661 Vairão, Portugal; 9 Estación Biológica de Doñana, Consejo Superior de Investigaciones Científicas (CSIC), Avda. Américo Vespucio 26, 41092 Sevilla, Spain; 10 BIOPOLIS Program in Genomics, Biodiversity and Land Planning, CIBIO, Campus de Vairão, 4485-661 Vairão, Portugal; 11 Departamento de Biologia, Faculdade de Ciências, Universidade Do Porto, 4099-002 Porto, Portugal; 12 Research Unit Modeling Nature, Universidad de Granada, 18071 Granada, Spain

**Keywords:** Computational biology and bioinformatics, Plant sciences

## Abstract

To explore the connection between chloroplast and coffee resistance factors, designated as S_H_1 to S_H_9, whole genomic DNA of 42 coffee genotypes was sequenced, and entire chloroplast genomes were de novo assembled. The chloroplast phylogenetic haplotype network clustered individuals per species instead of S_H_ factors. However, for the first time, it allowed the molecular validation of *Coffea arabica* as the maternal parent of the spontaneous hybrid “Híbrido de Timor”. Individual reads were also aligned on the *C. arabica* reference genome to relate S_H_ factors with chloroplast metabolism, and an in-silico analysis of selected nuclear-encoded chloroplast proteins (132 proteins) was performed. The nuclear-encoded thioredoxin-like membrane protein HCF164 enabled the discrimination of individuals with and without the S_H_9 factor, due to specific DNA variants linked to chromosome 7c (from *C. canephora*-derived sub-genome). The absence of both the thioredoxin domain and redox-active disulphide center in the HCF164 protein, observed in S_H_9 individuals, raises the possibility of potential implications on redox regulation. For the first time, the identification of specific DNA variants of chloroplast proteins allows discriminating individuals according to the S_H_ profile. This study introduces an unexplored strategy for identifying protein/genes associated with S_H_ factors and candidate targets of *H. vastatrix* effectors, thereby creating new perspectives for coffee breeding programs.

## Introduction

Coffee is the most important agricultural commodity, with more than 9 million tons consumed each year, and an estimated retail value of 70 billion US dollars. Coffee is crucial for the economy of more than 60 countries, and it is the primary source of income for more than 100 million people^1,2^. Its production is focused in developing countries, where coffee represents a substantial portion of export earnings and serves as a crucial source of livelihood for households^3^.

Even though the *Coffea* genus is estimated to contain around 120 species, coffee supply comes from two species: *Coffea arabica* and *Coffea canephora*, representing about 56% and 44% of the global production, respectively^3^. *Coffea arabica* is an allopolyploid species (*2n* = *4x* = 44) resulting from the natural hybridization between the diploid species (*2n* = *2x* = 22) *Coffea eugenioides* and *C. canephora*^4^. *Coffea arabica* shows low genetic diversity, climatic inelasticity, and susceptibility to diseases and pests^5–7^. On the other hand, *C. canephora* shows higher genetic diversity and better tolerance to adverse conditions such as high temperature, drought, and pathogen challenges^8^.

Coffee leaf rust (CLR) is one of the diseases most significantly affecting Arabica coffee production on a global scale^1^. It was first observed in 1861 on East African wild coffee plants, and in 1869 the biotrophic fungus *Hemileia vastatrix* was identified as its causal agent^1^. CLR disease causes premature leaf fall due to direct damage, weakening and favouring dieback of branches, decreasing the photosynthetic capacity and vigour of the infected coffee plants^1,9,10^. Due to the collapse of the coffee industry in several countries, efforts were made to identify and introduce coffee species with higher tolerance to CLR. *Coffea liberica* and *C. canephora* were among the earliest species to be introduced, resulting in the creation of numerous interspecific hybrids. The Kalimas and Kawisari hybrids (*C. arabica* × *C. liberica*) exhibited considerable variability and low productivity, while the spontaneous hybrid (*C. arabica* × *C. canephora*) known as “Híbrido de Timor” (HDT) proved to be more resilient^11^. HDT stands out as the most prominent interspecific hybrid, a tetraploid arabicoid found on Timor Island that exhibits heterogeneity in appearance and yield. The discovery of this hybrid with resistance to the main rust races was a breakthrough in the coffee breeding programs, which have been carried out by the Centro de Investigação das Ferrugens do Cafeeiro (CIFC), in Portugal over the last 50 years^11,12^ and references therein. HDT and its derivatives played a crucial role in controlled crosses alongside traditional *C. arabica* varieties. The breeding efforts led to the emergence of a wide array of progenies, and subsequently, commercial coffee varieties exhibiting strong rust resistance and high production. These resistant varieties have since been developed and made available in coffee-growing regions across Latin and Central America, Africa, and Asia^1,12^. The evidence of their widespread adoption is readily apparent through various available varieties catalogs, such as the World Coffee Research repository. The study of resistant inheritance on crosses involving HDT derivatives, several *C. arabica* varieties and other *Coffea* species led to the identification of at least nine rust resistance factors designated as S_H_1 to S_H_9. Those studies demonstrated that S_H_6-S_H_9 derives from *C. canephora* ancestors, S_H_3 from a *C. liberica* introgression and the remaining factors from *C. arabica*^11–14^ and references therein.

The arms race between plants and fungi includes multiple plant defence mechanisms that the pathogen continually tries to circumvent. A successful strategy from a biotrophic fungus’ point of view will be to control the host's primary metabolism for its own feeding purposes, while conversely, the plant may try to block the fungus' access. It was reported that in susceptible coffee plants, the *H. vastatrix* genes involved in sugar transport and metabolism were upregulated^15^. On the other hand, coffee plants treated with resistance inducers and challenged by *H. vastatrix* showed a reduced incidence of CLR disease which was related to primary metabolic adjustments, namely the up-regulation of proteins from the photosynthesis-related pathways and redox-related enzyme activities^16^. Coffee resistance to *H. vastatrix* has been associated with restricted fungal growth in the early stages of the infection process due to hypersensitive cell death (HR), accumulation of reactive oxygen species (ROS), haustoria encasement, and cell wall lignification^12^ and references therein. ROS retrograde signalling is involved in PTI (PAMP-Triggered Immunity) and ETI (Effector-Triggered Immunity) responses. The generation of the H_2_O_2_ signal in PTI occurs in photosystem I (PSI), while in ETI the H_2_O_2_ signal is generated under photosystem II (PSII). In both cases, there is a strong suppression of the nuclear-encoded chloroplast genes, including photosynthesis-related genes^17^. Considering the importance and interplay of ROS and carbohydrate metabolism to plant–pathogen interactions, the chloroplast represents a prime target for pathogens’ manipulation^18,19^. While the targeting of chloroplasts by effectors from filamentous pathogens is documented, and a dynamic role for the chloroplast metabolism in the regulation of immune responses is foreseen^18^, knowledge of chloroplast-localised rust effector proteins is very limited^17,20^. Chloroplast functioning can also be disturbed by cytosolic-acting effectors that block the translocation of chloroplast nuclear-encoded proteins from the cytosol to the chloroplast^21^.

In addition to the chloroplast’s role in plant immunity, and due to its maternal inheritance in the different coffee species and interspecific hybrids^22^ and references therein, the plastid genome (cpDNA) can also serve as a valuable tool for deducing ancestry and evolutionary relationships. The complete chloroplast genome of several coffee individuals has been described^23–31^. Genome annotation studies performed on *C. arabica* cpDNA have revealed the presence of a total of 114 unique genes, consisting of 80 protein-coding, 30 tRNA, and four rRNA genes^29^. Proteins encoded by *C. arabica c*hloroplast genomes are involved in photosynthesis (44 proteins), transcription (25 proteins), and functions such as protein degradation, fatty acid metabolism, and carbon fixation (6 proteins), with five more hypothetical reading frames of unknown function (*ycf1*–*ycf5*)^29^. The nuclear genome encodes all the remaining proteins required for chloroplast functions (including DNA replication, genome maintenance, and the regulation of gene expression and protein activity). Thus, most of the 2000–3000 proteins composing the chloroplast proteome are translated into the cytosol and imported into chloroplasts^32^. To assess whether there is a connection between chloroplast metabolism and coffee defence responses (S_H_ factors), we address two complementary questions:

1. Does the chloroplast genome reflect S_H_ phenotypes? To answer this question, we use chloroplast genome of 42 coffee genotypes from the CIFC collection with different resistance factors to *H. vastatrix*. We also make use of 18 conspecific genomes available at NCBI in 2022.08.
2. Do nuclear-encoded chloroplast proteins reflect S_H_ phenotypes? For this we performed an in-silico analysis of selected nuclear-encoded protein families acting on chloroplasts, focusing on gene families previously highlighted as being involved in *H. vastatrix* resistance^16,20,21^.

## Results and discussion

### Comparative chloroplast genomic analysis

**Figure 1:**
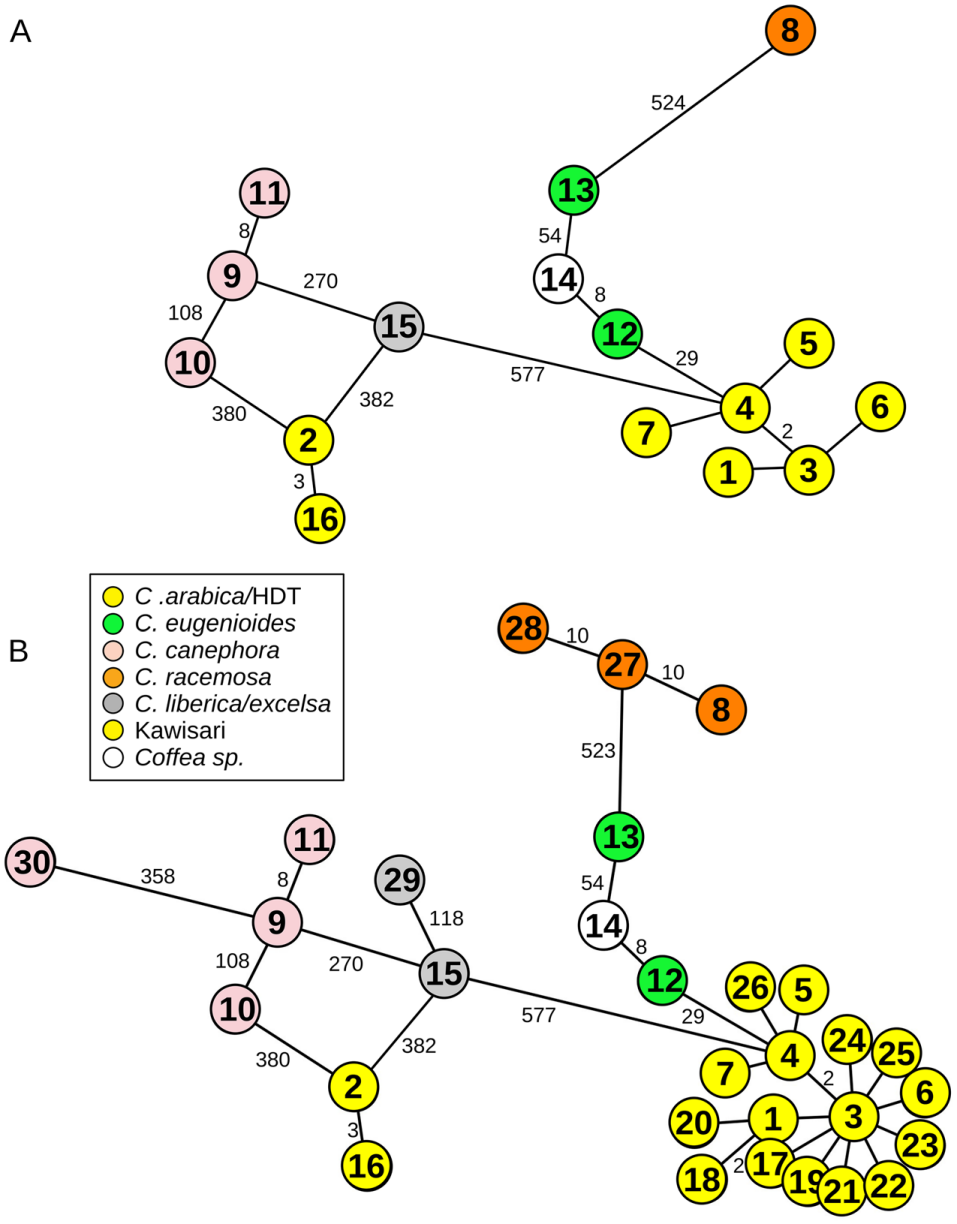
Haplotype network based on whole chloroplast genomes. (**A**) Newly assembled chloroplast genomes of 42 *Coffea* sp. genotypes. (**B**) Sixty *Coffea sp.* genotypes including the 18 conspecific individuals downloaded from GenBank. Numbers within circles identify the haplotypes. Numbers in edges indicate genetic distances higher than one. Network topology reflects genealogical relationships instead of rust resistance phenotype. Genotype and S_H_ details are presented in Supplemental Table S1.

To address whether cpDNA reflects S_H_ resistance phenotypes, we assembled the whole chloroplast genomes of 42 individuals and studied the topology of the resulting haplotype network. Newly assembled chloroplast genomes ranged between 154,815 and 155,188 bp long and were grouped into 16 haplotypes (Fig. 1A, Supplemental Table S1).

**Figure 2:**
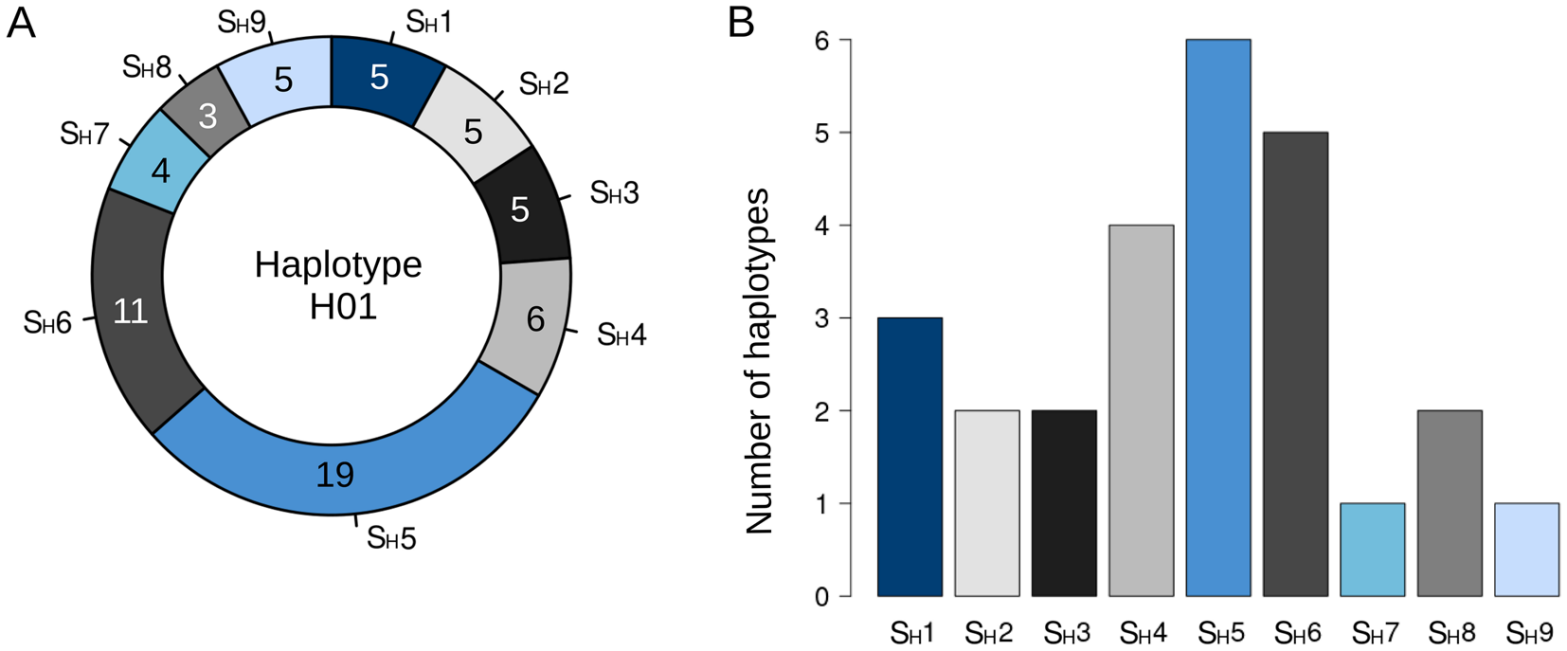
Lack of relationship between chloroplast haplotypes and S_H_ factors was confirmed as every haplotype is composed of individuals containing multiple S_H_ factors and every S_H_ factor appears in multiple haplotypes: (**A**) Number of S_H_ factors found in individuals showing Haplotype H01. Note that every individual may harbour more than one S_H_ factor. (**B**) Number of haplotypes containing each S_H_ factor. Only S_H_7 and S_H_9 were found in only one haplotype (H01).

Haplotype H01 was the most common haplotype, consisting of 22 individuals, with a high diversity of S_H_ factors (Supplemental Table S1). Indeed, all nine S_H_ factors were represented in this haplotype at least three times, such as S_H_8, and up to 19 times in the case of S_H_5 (Fig. 2A). Additionally, seven out of the nine S_H_ factors were found in two or more haplotypes (Fig. 2B). These results suggests a lack of maternal inheritance of the S_H_ resistance factors throughout the chloroplast genome. Consistently, haplotypes were distributed throughout the network, separating individuals per species instead of per S_H_ resistance factors.

Most *C. arabica,* HDT hybrids, and HDT-derivatives haplotypes clustered together (exceptions being haplotypes H02 and H16, Fig. 1A). This low differentiation was not surprising given that most of the *C. arabica* and HDT-derivative individuals share close kinship (Supplemental Table S1). Examples can be found in haplotype H03, which included the parental female *C. arabica* Dilla and Alghe (CIFC 128/2) and the sibling HDT derivative H468/41 (*C. arabica* 128/2 × HDT 1343/269); haplotype H05, which included the parental female *C. arabica* S4 Agaro (CIFC 110/5) and the sibling HDT derivative H583/5 (*C. arabica* 110/5 × HDT 1343/269). Furthermore, some of the closest haplotypes included individuals that come from the same geographic origin, such as H04 and H05 (with Ethiopian backgrounds; Supplemental Table S1). Others, like haplotypes H03 and H07, grouped landrace genotypes from the northeast African highlands (Rume Sudan Ethiopian landrace; Supplemental Table S1), the geographic origin of *C. arabica*^11,14^. When the information from 18 conspecific individuals from NCBI (13 *C. arabica* individuals) was included in the haplotype network, the Arabica cluster was reinforced (Fig. 1B). *Coffea arabica*, HDT hybrids, and HDT-derivatives cluster was closer to *C. eugenioides* haplotypes (genetic distance = 29, Fig. 1B) than to *C. canephora* haplotypes (genetic distance higher than 800). This was congruent with the accepted hypothesis that *C. eugenioides* was the female parent of *C. arabica*^4,6^. Indeed, based on the similarity in plastid DNA sequences, previous research has suggested that *C. eugenioides* was the ovule donor during the *C. arabica* hybridization event^7,33–35^. Our analysis further contributes to the maternal lineage of *Coffea* sp. (CIFC 951/1; haplotype H14). The position of haplotype H14 in the network (between haplotypes H12 and H13) suggests a maternal inheritance close to *C. eugeneoides* or a near coffee species.

The chloroplast genome of the HDT hybrids analysed further suggested *C. arabica* as the female parent. All HDT hybrids were within the haplotype H01, with a high genetic distance from *C. canephora* genotypes (higher than 800, Fig. 1). To our knowledge, this was the first molecular study addressing the maternal donor of HDT as morphological characteristics were used to infer *C. arabica* × *C. canephora* as its ancestors^11,14^. However, it is worth noting that our approach does not allow us to infer about the initial hybridization event (*C. arabica* × *C. canephora*) just about the maternal donor of the CIFC HDT hybrids used in this work. Further research is needed to address this and other alternative hypotheses.

Our analyses also revealed a very low genetic distance between individuals from haplotypes H02 and H16, suggesting a close maternal parentage between them (Fig. 1). Haplotype H16 represented the Kawisari hybrid, one of the oldest Indian hybrids created from *C. arabica* × C*. liberica*; haplotype H02 comprised three kinship individuals: *C. arabica* S 353 4/5 (CIFC 34/13), the female parental of both *C. arabica* H147/1 (*C. arabica* 34/13 × *C. arabica* 110/5) and HDT derivative H535/10 (*C. arabica* 34/13 × HDT 1343/269) (Fig. 1, Supplemental Table S1). The *C. arabica* S 353 4/5 genotype¸ from the Central Coffee Research Institute (CCRIBalehonnur, India) was originated from a selection of *C. arabica* × *C. liberica* derivatives^11^. We found a substantial genetic distance between haplotype H02 and the other Arabica haplotypes (almost one thousand). Indeed, haplotypes H02 and H16 were more closely related to *C. canephora* and *C. liberica*/*excelsa* haplotypes (genetic distance = 380 and 382, respectively; Fig. 1B). These findings raised the possibility that the genotypes within H02 and H16 may have originated from a non-*arabica*/*eugenioides* female parent. Further research is needed to ascertain its inheritance.

Haplotypes found in *C. canephora*, *C. liberica*/*excelsa*, *C. racemosa,* and *C. eugenioides* showed considerable genetic distances within each species, particularly when compared with *C. arabica* (Fig. 1)*.* Our data reinforced the knowledge of the lower polymorphic genetic diversity of *C. arabica* when compared to the diploid relative species^36^.

### Comparative analysis of nuclear genes encoding chloroplast-targeted proteins

**Figure 3:**
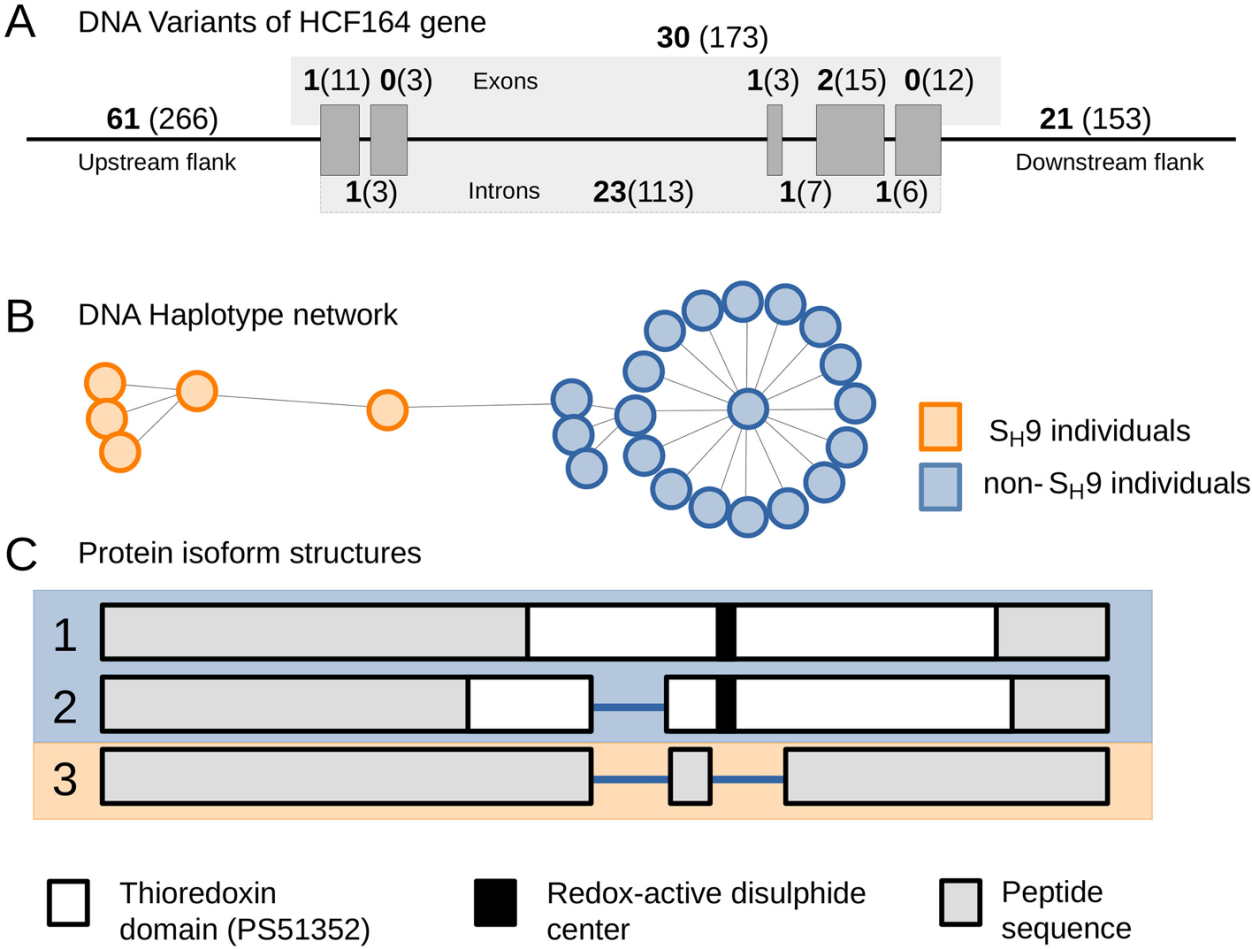
Analysis of the HCF164 sequence in chromosome 7c (*C. canephora*-derived sug-genome): (**A**) Depiction of the variants identified in the genomic region encoding the HCF164 protein in chromosome 7c following the reference genome annotation (GCA_003713225). The diagram shows the ORF (composed of five exons represented as grey rectangles) and 2 kbp upstream and downstream flanking regions. The numbers in brackets represent the number of variants identified in the 25 studied individuals, whereas the numbers in bold represent variants potentially associated with the S_H_9 factor (that is, variants exclusively found in S_H_9 individuals). (**B**) Haplotype network of the genomic region encoding the HCF164 protein (including 2 kbp flanking regions) obtained for the 25 studied individuals. (**C**) Schematic view of the alignment of the three HCF164 protein isoforms predicted in the 25 studied individuals. The thioredoxin domain and the redox-active disulphide center are highlighted in white and black, respectively. Orange represents S_H_9 individuals and blue represents non-S_H_9 individuals.

After confirming that cpDNA did not explain individual resistance patterns, we focused on nuclear-encoded chloroplast proteins, described as targets of retrograde signalling generated within the chloroplast^17^. The chloroplast proteome has been estimated between 2100 and 3600 proteins, and approximately 3000 chloroplast proteins are nuclear-encoded^37^. To detect possible association with S_H_ factors, we focused on the 25 individuals with known S_H_ factors (Supplemental Table S1) and on the following nuclear-encoded protein families involved in resistance and acting on chloroplasts^16,20,21^: ATP-dependent zinc metalloprotease (FtsH); Elongation factor Tu (EFTU); Ferredoxin-thioredoxin reductase (FTR); Thioredoxin reductase (TRR); d-glycerate 3-kinase (GLYK); NAD(P)H dehydrogenase-like (NDH); Thioredoxin and Thioredoxin-like (TRX); Translation initiation factor (IF); Oxygen-evolving enhancer protein (OEE); and Cytochrome *b*_*6*_*-f* complex iron-sulphur subunit (ISP). In total, 132 nuclear-encoded chloroplast proteins associated with 89 nuclear regions were analysed considering DNA variants in the ORF as well as upstream and downstream flanking regions (Supplemental Table S2). We found 139 variants unevenly distributed among 11 nuclear regions that corresponded to polymorphisms in 8 proteins (Table 1). In addition, several variants found in the upstream and downstream flanking regions (regions 61 and 21 in Fig. 3A, respectively) can also play a role in controlling transcriptional and post-transcriptional events. A disproportionate number of variants for the membrane-anchored thioredoxin-like protein HCF164 (114 out 139) were exclusively shared among S_H_9 individuals and were mainly associated with the *C. canephora-*derived sub-genome (chromosome 7c, 112 variants) (Fig. 3A, Table 1). A detailed analysis of this gene showed clear differences between individuals with or without the S_H_9 factor (Fig. 3B,C). The clustering of individuals within the haplotypic network estimated for this region suggested the potential relationship between variants identified in the HCF164 nuclear region and the presence of the S_H_9 factor (Fig. 3B). Moreover, the observed variants impacted the peptide sequence codified by this region as protein prediction performed on the 25 studied individuals allowed us to identify three HCF164 protein isoforms. Two of the previous isoforms were exclusively found in non-S_H_9 individuals both exhibiting the thioredoxin domain and the redox-active disulphide center (CEVC catalytic motif). On the other hand, the five S_H_9 individuals (HDT genotypes: 832/1; 4106; H420/10; HW26/13; H419/20) shared the third isoform, in contrast to those found in the non-S_H_9 individuals. This isoform lacks the thioredoxin domain and the peptide sequence of the redox-active disulphide center due to a 19-residue deletion identified in this work (Fig. 3C).

**Table 1:** DNA variants of chloroplast nuclear-encoded proteins potentially associated with S_H_ phenotypes.

Chromosome	Locus code	Protein description	S_H_	S_H_-associated variants			Number of variants per *locus*
				Upstream	ORF	Downstream	
Chromosome 2c	LOC113725880	Thioredoxin-like fold domain-containing protein MRL7 homolog chloroplastic isoform X1	8	7			7
Chromosome 2e	LOC113730788	Thioredoxin-like 3–2 chloroplastic isoform X1	8			4	4
Chromosome 3c	LOC113735166	Translation initiation factor IF-2 chloroplastic-like isoform X2	3			1	1
Chromosome 4e	LOC113742584	d-glycerate 3-kinase, chloroplastic isoform X1	9	1	5		6
Chromosome 6c	LOC113691470	Thioredoxin reductase NTRC-like	7	1			1
Chromosome 6c	LOC113692086	Thioredoxin-like 3–1 chloroplastic	7	2			2
Chromosome 6c	LOC113692806	Putative elongation factor TypA-like SVR3	7		1		1
Chromosome 7c	LOC113700207	Thioredoxin-like protein HCF164 chloroplastic	7			2	2
			9	61	30	21	112
Chromosome 7e	LOC113700346	Thioredoxin-like protein HCF164 chloroplastic	7			1	1
			9			2	2

Chromosome number and parental origin (c—*C. canephora*; e—*C. eugenioides*), *locus* code, protein description, S_H_ factor and number of variants found in the ORF and the upstream and downstream flanking regions are shown.

Three-dimensional structural models were developed for HCF164 proteins expressed in S_H_9 and non-S9 individuals. The α-helix ranging from cysteine 163 to aspartate 182 (C163–D182) containing the typical CEVC catalytic motif of the protein was completely absent in the S_H_9-individuals (Supplemental Figure S1). This suggests that the lack of the redox-active disulphide center of HCF164 protein in the S_H_9 individual might have important biochemical implications as thioredoxins target several proteins and can modulate their activity. HCF164 is a membrane-anchored thioredoxin-like protein known to be indispensable for the assembly of the cytochrome *b*_*6*_*-f* complex (Cyt*b*_*6*_*-f*) in the thylakoid membranes; the loss-of-function *hcf* mutants exhibited decreased photosynthetic electron transport rates^38^.

Cyt*b*_*6*_*-f* provides an essential electronic connection between the light-powered chlorophyll protein complexes, photosystems I and II (PSI and PSII). It is suited to sensing the redox state of the electron transfer chain and the chloroplast stroma, interacting with various regulatory elements that transduce these signals to optimise photosynthesis in fluctuating environmental and metabolic conditions^39^. Cyt*b*_*6*_*-f* complex is a ~ 220 kDa functional dimer with each monomeric unit comprising four major subunits: cytochrome *f*, cytochrome *b*_*6*_, Rieske iron-sulphur protein (ISP) and subunit IV; as well as four minor subunits^39^ and references therein. Results obtained by Motohashi and Hisabori^40^ suggested that the interaction between HCF164 and both the cytochrome *f* and ISP subunits were important prerequisites for the correct assembly of the Cyt*b*_*6*_*-f* complex. They further evidenced the physiological significance of HCF164 as a transducer of reducing equivalent within the thylakoid lumen. In addition to this complex, HCF164 may interact and probably reduce other target proteins of the thylakoid membrane, such as metalloprotease FtsH2 and FtsH8, several ATP synthase subunits and chlorophyll a-b binding proteins^38,40^.

**Figure 4:**
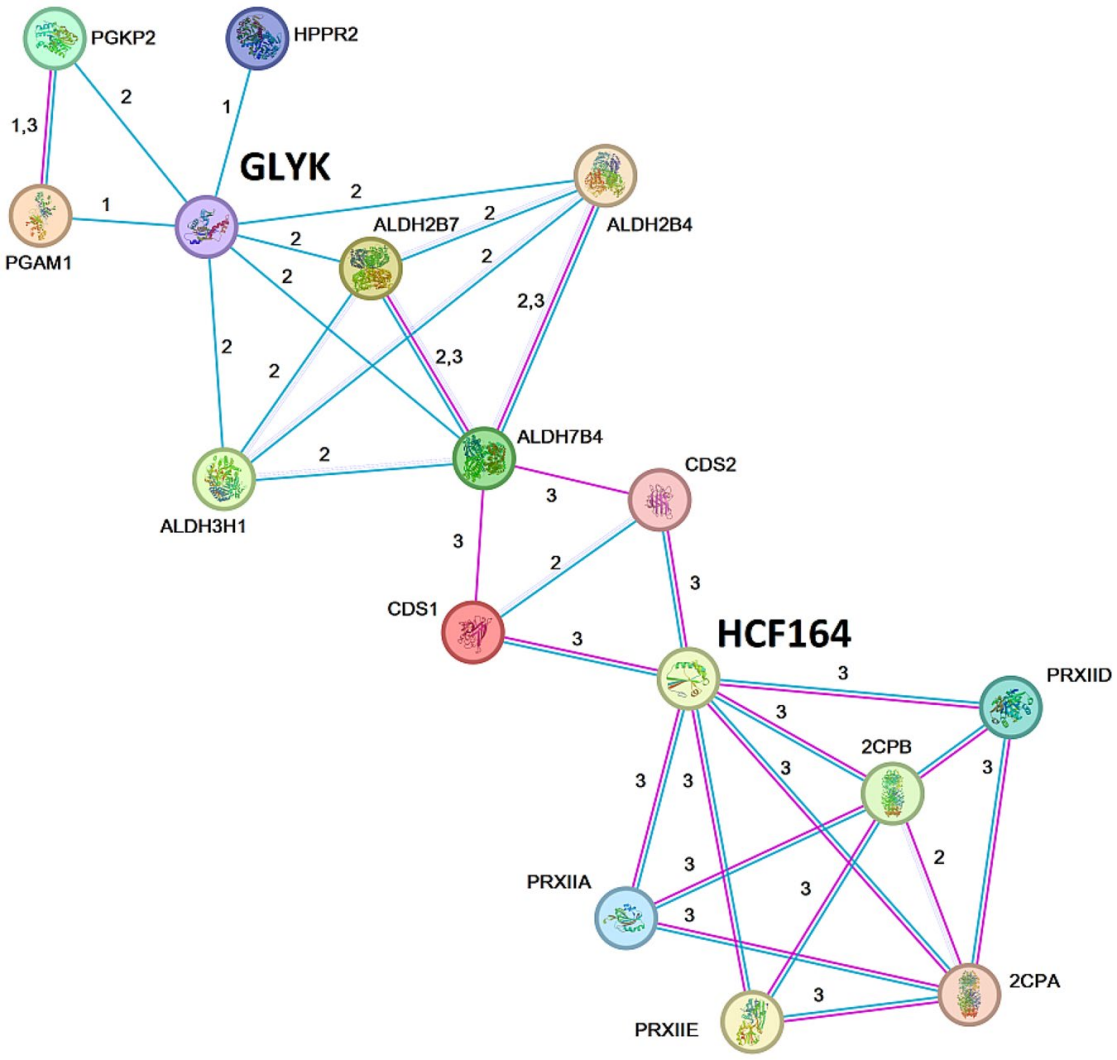
String predicted network for Arabidopsis thioredoxin-like protein (HCF164, AT4G37200) and d-glycerate 3-kinase (GLYK, AT1G80380) interaction. Only “Experiments” and “Databases” as active interaction sources and “none” in the 1st and 2nd shells were considered. Blue edges—evidence from curated databases; Pink edges—experimental evidence. Numbers: 1—evidence suggesting a functional link; 2—evidence suggesting a functional link and putative homologs were found interacting in other organisms; 3—putative homologs were found interacting in other organisms. Proteins included were: Phosphoglycerate kinase (PGKP2, AT1G56190); Phosphoglycerate mutase (PGAM1, AT1G22170); Glyoxylate/hydroxypyruvate reductase (HPPR2, AT1G79870); Glyceraldehyde-3-phosphate dehydrogenases (ALDH3H1, AT1G44170; ALDH7B4; ALDH2B4, AT3G48000; ALDH2B7); Superoxide dismutase [Cu–Zn] 1 and 2 (CSD; AT1G08830, AT2G28190); Peroxiredoxin II A, D and E (PRXII; AT1G65990, AT1G60740, AT3G52960) and 2-Cys-peroxiredoxin A and B (2CP; AT3G11630, At5g06290).

HCF164 protein–protein interactions were explored with the STRING database (only protein–protein interactions retrieved from Experimental/Biochemical Data or Association in Curated Databases were considered) using Arabidopsis protein annotations (as the interaction networks are better characterised than in coffee). As DNA variants for GLYK (6 variants localised in chromosome 4e; Table 1) were also exclusively found in S_H_9 individuals, we consider both proteins for the STRING analysis. Although no direct interaction between HCF164 and GLYK proteins was evidenced, the enrichment *p*-value obtained (< 1.0e−16) supports that, as a group, the proteins were metabolically connected (Fig. 4) through redox metabolism, photorespiration, and glycolysis. GLYK catalyses the conversion of glycerate to 3-phosphoglycerate involved in photorespiration and redox metabolism. The glyceraldehyde-3-phosphate dehydrogenases ALDH7B4 and ALDH3H1 are described as stress-responsive dehydrogenases that catalyse the conversion of glyceraldehyde 3-phosphate to d-glycerate 1,3-bisphosphate. HCF164 shows several interactions with superoxide dismutase (CDS1, CDS2) and peroxiredoxins (2CPA, 2CPB, PRXIIA, PRXIID, PRXIIE) (Fig. 4). Thereby, any changes to the balance of these proteins can affect chloroplast metabolism.

Recently, the mechanisms of stripe rust (*Puccinia striiformis* f. sp. *tritici*) effectors in wheat have been identified. Rust effectors targeted the ISP subunit of the Cyt*b*_*6*_*-f* complex: some effectors interacted with ISP (nuclear-encoded chloroplast protein) in the cytosol blocking its translocation to the chloroplast^21^; other effectors interacted with ISP within the chloroplast preventing the complex assembly^20^. Both types of effectors interfered with the Cyt*b*_*6*_*-f* complex functioning and ROS production by chloroplasts^20,21^. The authors further showed that completely blocking the Cyt*b*_*6*_*-f* complex assembly was not advantageous for the fungus as it led to insufficient nutrients for fungal development in the latter stages of infection. So, although biotrophic rust fungi need to suppress chloroplast-mediated defences by their host plants, they need to retain the biosynthetic abilities of these organelles, which are vital for their survival.

The association of HCF164 polymorphism on chromosome 7c (*C. canephora*-derived sub-genome) with the resistance factor S_H_9 aligns with the existing information that the S_H_9 coffee resistance factor to *H. vastatrix* is derived from major genes from *C. canephora* (considered a resistance source)^12^ and references therein. The lack of a thioredoxin domain and redox-active disulphide center of HCF164 protein isoform expressed only by the S_H_9 individuals may suggest a biochemical advantage of these individuals over others. This difference in HCF164 function may result in a greater ability for S_H_9 individuals to resist fungal infections or to better regulate other biological processes. On the other hand, the redox-related roles of HCF164 might be taken over by other thioredoxins-like proteins. It will be necessary to determine if S_H_9-HCF164 is recognized by *H. vastatrix *effectors and if it could act as a decoy, preventing the effector’s function(s) while still allowing normal plant development. However, functional redundancy (or metabolic flexibility) is proposed but has not yet been fully characterised. Our results further reinforce the chloroplast-mediated defences against leaf rust, particularly carbon metabolism and redox homeostasis^16^. This study shows a strategy for searching proteins/genes associated with S_H_ factors as well as candidate *H. vastatrix* effector targets, thus opening new perspectives for plant breeding programs.

## Material and methods

### Biological material

Forty-two coffee genotypes, including HDT, HDT-derivatives, *C. arabica*, *C. eugenioides*, *C. canephora*, and other related *Coffea* sp. were used (Table 2). Genotypes with different coffee resistance factors to *H. vastatrix* (from highly susceptible to highly resistant to rust) were considered. This selection was performed to maximise the number of individuals showing every known S_H_ while minimising the total number of individuals (n = 25). Additional eighteen individuals carrying unknown S_H_ factors were included to reconstruct the evolutionary and ancestry patterns among the studied individuals (Supplemental Table S1).

**Table 2:** Coffee genotypes from the CIFC collection used in this study, with information on geographic origin and S_H_ factor.

Coffee genotypes	CIFC number	SH factor	Origin
*Coffea* sp.	951/1	–	Dem. Rep. Congo
*C. racemosa*	13,969	–	Mozambique
*C. canephora*	829/1	–	Angola
*C. canephora*	2975	–	Angola
*C. canephora*	1459	–	Indonesia
*C. eugenioides*	16,486/23	–	Brazil
*C. eugenioides*	214/43	–	Tanzania
*C. excelsa*	51/5	–	Madagascar
Kawisari hybrid	644/18	S_H_?	Indonesia
HDT hybrid	832/1	S_H_5,6,7,8,9,?	Timor
HDT hybrid	4106	S_H_5,6,7,8,9,?	Timor
HDT hybrid	1343/269	S_H_6	Timor
HDT hybrid	1343/252	S_H_?	Timor
HDT hybrid	19,129/4	S_H_?	Timor
HDT hybrid	19,138/6	S_H_?	Timor
HDT hybrid	2252/28	S_H_?	Timor
*C. arabica* Matari	849/1	S_H_?	Yemen
*C. arabica* Caturra	19/1	S_H_5	Angola
*C. arabica* Bourbon	63/1	S_H_5	Brazil
*C. arabica* Rume Sudan	21,336/4	S_H_5	Kenya
*C. arabica* DK 1/6	32/1	S_H_2,5	India
*C. arabica* S 288–23	33/1	S_H_3,5	India
*C. arabica* Dilla and Alghe	128/2	S_H_1	Kenya
*C. arabica* S 353 4/5	34/13	S_H_2,3,5	India
*C. arabica* S 12 Kaffa	134/4	S_H_1,4	Ethiopia
*C. arabica* S 4 Agaro	110/5	S_H_4,5	Ethiopia
*C. arabica* S 12 Kaffa	635/3	S_H_1,4,5	Ethiopia
*C. arabica*	H632/16	S_H_3	CIFC
*C. arabica*	H147/1	S_H_2,3,4,5	CIFC
*C. arabica*	H426/2	S_H_5	CIFC
*C. arabica*	H426/2-141-11	S_H_?	CIFC
HDT derivative	HW 26/13	S_H_5,6,7,9,?	CIFC
HDT derivative	H419/20	S_H_5,6,9	CIFC
HDT derivative	H420/2	S_H_5,8	CIFC
HDT derivative	H420/10	S_H_5,6,7,9	CIFC
HDT derivative	H537/18	S_H_2,5,6	CIFC
HDT derivative	H468/41	S_H_1,6	CIFC
HDT derivative	H535/10	S_H_2,3,5,6	CIFC
HDT derivative	H539/8	S_H_1,4,6	CIFC
HDT derivative	H583/5	S_H_4,5,6	CIFC
Cavimor	13,727/18	S_H_?	Costa Rica
Cavimor	13,726/20	S_H_?	Costa Rica

S_H_?—verified resistance factor but unknown gene number.

### DNA extraction, sequencing, and nuclear genome analysis

For every individual, we isolated DNA from fresh leaf tissue using the NucleoSpin Plant II kit (Macherey–Nagel) following the manufacturer’s instructions. We prepared libraries using the TruSeq DNA PCR-Free kit (Illumina), and sequencing was performed by Novogene Inc. using two lanes of the HiSeq × System (Illumina) using 2 × 150 bp paired-end reads. We used CUTADAPT^41^ to filter the resulting fastq files and retained fragments with quality values higher than 20. We aligned filtered reads using BWA^42^ with default parameters and the *C. arabica* genome (GenBank accession number GCA_003713225.1) as a reference. We used SAMtools^43^ and BCFtools^44^ to process mapped reads, perform variant calls, subset vcf files, and obtain fasta DNA sequences per candidate region.

### Chloroplast genome analysis

#### De novo assembly

We used NOVOPlasty^45^ to assemble individual chloroplast genomes from raw fastq files. We used the large subunit of the ribulose-1,5-bisphosphate carboxylase/oxygenase (RuBisCO) from *Zea mays* (GenBank accession number V00171.1) as a seed and the entire chloroplast of *C. arabica* (EF044213.1) as a reference to solve conflicting regions found during the assembly. Following this strategy, we obtained circularised molecules for all 42 individuals that were subsequently reset using custom R scripts to homogenise the starting position to the sequence to TAGGCGAACGACGGGAATTGAA (one mismatch allowed). This sequence (corresponding with the intergenic region between *trnH*-*GUG* and *rps19*) was selected because it is the starting point in several *C. arabica* chloroplast genomes available in GenBank. The resulting complete chloroplast genomes are available in GenBank with accession numbers OQ946685-OQ946726 and were aligned using MAFFT^46^ with the “—auto” flag. For haplotype analysis, and in addition to the genomes of the 42 CIFC individuals we made use of 18 *Coffea* sp. chloroplast genomes available in GenBank (see details below).

We identified haplotypes on the aligned sequences using the haplotype function in the pegas package in R^47^, setting the argument “strict = TRUE” to consider ambiguities and gaps to differentiate haplotypes. We also used the pegas package to compute the haplotype network as implemented in the haploNet function with default arguments. Aliview v1.25^48^ was used to visualise fasta sequences.

#### Coffea chloroplast genomes available at the GenBank

We searched all the sequences in the GenBank Nucleotide database (https://www.ncbi.nlm.nih.gov/nuccore, accessed 19 August 2022) containing the word “*coffea*” and filtered the resulting sequences by genetic compartment (Chloroplast) and sequence length (100,000–200,000 bp). As a result, we obtained 32 accessions belonging to 17 species with genome sizes ranging from 154,545 to 155,277 bp. Only genomic information from species represented in 42 CIFC genotypes was further considered (18 conspecific individuals). Thirteen of the 18 accessions are classified as *C. arabica* which showed the largest genome sizes (ranging from 155,186 to 155,277 bp whereas the remaining species range from 154,751 to 154,951 bp, Supplemental Table S1).

### Nuclear-encoded candidate protein selection

In previous studies, several genes/proteins were identified as candidate coffee resistance markers that are simultaneously involved in chloroplast primary metabolism^16^. In addition, Xu et al.^20^ and Wang et al.^21^ highlighted the suppression of chloroplast function by wheat stripe rust effectors, targeting the cytochrome *b*_*6*_*-f* complex and, thus the photochemical reactions. Considering the information provided by these studies, we considered ten nuclear-encoded chloroplast protein families as potential candidates involved in coffee rust resistance. The selected protein families were ATP-dependent zinc metalloprotease (FtsH); Elongation factor Tu (EFTU); Ferredoxin-thioredoxin reductase (FTR); Thioredoxin reductase (TRR); d-glycerate 3-kinase (GLYK); NAD(P)H dehydrogenase-like (NDH); Thioredoxin and Thioredoxin-like (TRX); Translation initiation factor (IF); Oxygen-evolving enhancer protein (OEE); and Cytochrome *b*_*6*_*-f* complex iron-sulphur subunit (ISP).

Using the *C. arabica* gff3 file (available at https://www.ncbi.nlm.nih.gov/genome/browse/#!/proteins/77/418079%7CCoffea%20arabica/, last accessed on 4 November 2022), we identified all the proteins with annotation matching the following terms: “Ferredoxin-thioredoxin reductase” (4 proteins), “Oxygen-evolving enhancer protein” (4 proteins), “thioredoxin” (113 proteins found, used 58), “thioredoxin reductase” (4 proteins), “NAD(P)H dehydrogenase” (18 proteins), “glycerate” (6 proteins found, used 2), “FtsH” (26 proteins found, used 18), “elongation factor” (74 proteins found, used 10), “translation initiation factor” (142 proteins found, used 13), “b6-f” (1 protein found). Only DNA nuclear-encoded proteins were considered and, overall, our search resulted in a total of 132 proteins associated with 89 different *loci* (Supplemental Table S2). We used the *C. arabica* gff3 to record the starting and ending position of every protein gene within the *C. arabica* genome. We obtained fasta files for every Open Reading Frame (ORF) using BCFtools^44^ and the vcf file resulting from aligning our sample genomes to the reference as described above. In addition, we produced another two fasta files, one containing 2000 bp upstream of the first nucleotide in the ORF and the other containing 2000 bp downstream of the last nucleotide in the ORF. For every of the three resulting fasta files (that is, the ORF and the two flanking regions) we used a custom R script to identify variants exclusively shared among individuals showing a given S_H_. Only genotypes with known S_H_ (n = 25) were considered for this analysis, aiming to associate single-nucleotide polymorphisms (SNPs) profiles with a particular S_H_.

### Nuclear candidate protein isoforms

SNPs and other variants identified in a sequence may impact the primary sequence of amino acids, not only by changing the codon in a triplet but also by changing nucleotides defining introns and exons or modifying regulatory sequences. To evaluate the potential impact of variants exclusively shared among individuals showing a given S_H_, we performed gene prediction on individual sequences as implemented in the AUGUSTUS web interface (available at: http://bioinf.uni-greifswald.de/augustus/submission.php^49^) using the training for *Solanum lycopersicum* and selecting the options “Report genes on both strands”, “Middle alternative transcripts”, and “only predict complete genes”. We searched for conserved domains in every resulting predicted peptide using the Expasy ScanProsite tool (available at https://prosite.expasy.org/scanprosite/) and DELTA-BLAST (Domain Enhanced Lookup Time Accelerated BLAST available at https://blast.ncbi.nlm.nih.gov/Blast.cgi?PROGRAM=blastpandPAGE_TYPE=BlastSearchandLINK_LOC=blasthome).

### Nuclear candidate protein interaction network

Our analyses highlight two proteins, the thioredoxin-like protein HCF164 (Supplemental Table S2, protein identifier TRX44) and the d-glycerate 3-kinase isoform X1 (Supplemental Table S2, protein identifier GLYK2), as having different SNP profiles for genotypes showing and lacking S_H_9. To explore their potential effect on metabolic pathways, we reconstructed the interaction network of those proteins using STRING v1.5 (https://string-db.org/; 24 May 2022). The STRING database^50^ allows for the exploration of known and predicted protein–protein interaction networks, particularly in well-characterised model species, such as *Arabidopsis thaliana*. The analysis was performed using the *A. thaliana* homologs (AT4G37200.1 and AT1G80380.2 for HCF164 and GLYK, respectively), identified by the amino acid sequence of our candidate proteins from *Coffea* (using the “search protein by sequence” STRING tool). The interaction network for each protein was obtained using the following settings: “Experiments” and “Databases” as active interaction sources; “0.400” as the minimum required interaction score (default); “no more than 20 interactions” in the 1st and 2nd shell. Proteins retrieved as having an interaction with HCF164 or GLYK were used to generate a protein–protein interaction network (same settings applied but with “none” in the 1st and 2nd shells).

### HCF164 protein molecular modelling

A tridimensionality structural model for the thioredoxin-like domain (from G131 to E244) containing the CEVCRELAPDIYKIEQQYK deletion was built for the HCF164 protein using as a template for the high confidence regions, namely from T104 to V253, of the Alpha fold structure for *C. arabica* (https://www.uniprot.org/uniprotkb/A0A6P6TDQ6/entry). One hundred models were generated with the Modeller 9v22^51^, and the one with the lowest value for Modeller’s objective function was selected and validated using Procheck^52^. All molecular figures were created using PyMOL^53^.

### Research Plant Statement

All methods were carried out in accordance with relevant institutional, national, and international guidelines and legislation guidelines complying with the Convention on Biological Diversity (https://www.cbd.int/convention/) and the Convention on the trade in Endangered Species of Wild Fauna and Flora (https://cites.org/eng). All plants used in this study belong to Centro de Investigação das Ferrugens do Cafeeiro CIFC—Coffee Rust Research Center) collection. CIFC is a research and advanced training Center of the Instituto Superior de Agronomia (ISA), Universidade de Lisboa (ULisboa) (https://www.isa.ulisboa.pt/en/cifc/about). The work presented in the manuscript was approved and developed under the research agreement of HDT-Coffee project.

### Supplementary Information

Supplementary Information.

## Data Availability

Individual chloroplast genomes generated for this study can be found in GenBank Accession Numbers OQ946685-OQ946726.
