# Publisher Correction: The chloroplast protein HCF164 is predicted to be associated with *Coffea* S_H_9 resistance factor against *Hemileia vastatrix*

**DOI:** 10.1038/s41598-023-45266-1

**Published:** 2023-10-25

**Authors:** Leonor Guerra-Guimarães, Carla Pinheiro, Ana Sofia F. Oliveira, Andrea Mira-Jover, Javier Valverde, Fernanda A. de F. Guedes, Herlander Azevedo, Vitor Várzea, Antonio Jesús Muñoz Pajares

**Affiliations:** 1 CIFC - Centro de Investigação das Ferrugens do Cafeeiro, Instituto Superior de Agronomia, Universidade de Lisboa, Tapada da Ajuda, 1349-017 Lisboa, Portugal; 2 LEAF - Linking Landscape, Environment, Agriculture and Food Research Center, Associated Laboratory TERRA, Instituto Superior de Agronomia, Universidade de Lisboa, Tapada da Ajuda, 1349-017 Lisboa, Portugal; 3 UCIBIO Applied Molecular Biosciences Unit, Department of Life Sciences, NOVA School of Science and Technology, Universidade NOVA de Lisboa, 2829-516 Caparica, Portugal; 4 Associate Laboratory i4HB Institute for Health and Bioeconomy, NOVA School of Science and Technology, Universidade NOVA de Lisboa, 2829-516 Caparica, Portugal; 5 Center for Computational Chemistry, School of Chemistry, University of Bristol, University Walk, Bristol, BS8 1TS UK; 6 Departamento de Genética, Universidad de Granada, 18071 Granada, Spain; 7 Área de Ecología, Departamento de Biología Aplicada, Universidad Miguel Hernández, Elche, Spain; 8 CIBIO, Centro de Investigação em Biodiversidade e Recursos Genéticos, InBIO Laboratório Associado, Universidade do Porto, Campus de Vairão, 4485-661 Vairão, Portugal; 9 Estación Biológica de Doñana, Consejo Superior de Investigaciones Científicas (CSIC), Avda. Américo Vespucio 26, 41092 Sevilla, Spain; 10 BIOPOLIS Program in Genomics, Biodiversity and Land Planning, CIBIO, Campus de Vairão, 4485-661 Vairão, Portugal; 11 Departamento de Biologia, Faculdade de Ciências, Universidade Do Porto, 4099-002 Porto, Portugal; 12 Research Unit Modeling Nature, Universidad de Granada, 18071 Granada, Spain

Correction to: *Scientific Reports* 10.1038/s41598-023-41950-4, published online 25 September 2023

The original version of this Article contained an error in Figure 3, panels B and C, where the results were incorrectly displayed.

**Figure 3:**
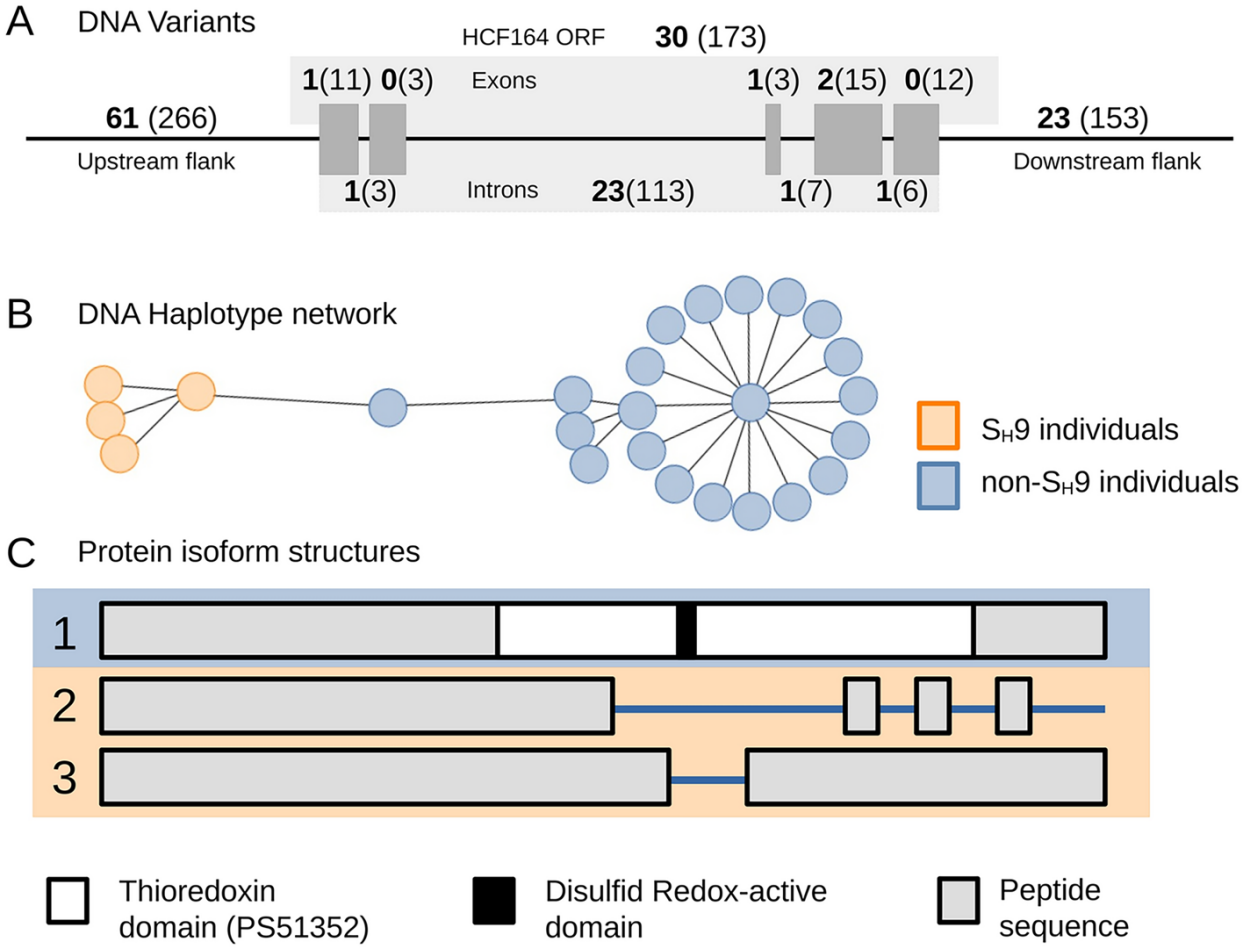
Analysis of the HCF164 sequence in chromosome 7c (*C. canephora*-derived sug-genome): (**A**) Depiction of the variants identified in the genomic region encoding the HCF164 protein in chromosome 7c following the reference genome annotation (GCA_003713225). The diagram shows the ORF (composed of five exons represented as grey rectangles) and 2 kbp upstream and downstream flanking regions. The numbers in brackets represent the number of variants identified in the 25 studied individuals, whereas the numbers in bold represent variants potentially associated with the SH9 factor (that is, variants exclusively found in SH9 individuals). (**B**) Haplotype network of the genomic region encoding the HCF164 protein (including 2 kbp flanking regions) obtained for the 25 studied individuals. (**C**) Schematic view of the alignment of the three HCF164 protein isoforms predicted in the 25 studied individuals. The thioredoxin domain and the redox-active disulphide center are highlighted in white and black, respectively. Orange represents SH9 individuals and blue represents non-S_H_9 individuals.

The original Article has been corrected.

