# Supplementary material for: The chloroplast protein HCF164 is predicted to be associated with Coffea SH9 resistance factor against Hemileia vastatrix
Source: Sci Rep. 2023 Sep 25;13:16019. doi: 10.1038/s41598-023-41950-4 (PMC10520047; doi:10.1038/s41598-023-41950-4)
Supplement: Supplementary file 1 — Supplementary Information. [file 41598_2023_41950_MOESM1_ESM.pdf]

Table S1. Chloroplast genome haplotypes for the 61 *Coffea* sp. genotypes from CIFC collection and NCBI GenBank database. Colour code in the haplotype column stands for chloroplast haplotype network shown in Figure 1. Available information on geographic origin, S_H factor and descendants associated with each genotype are provided.

Haplotype	Genome length (bp)	Coffee species/variety	Genotype Identifier	S_H factor	Origin	Notes
1	155188	HDT hybrid	CIFC: 832/1	S_H5,6,7,8,9,?	Timor	(*C. arabica* x *C. canephora*)
		HDT hybrid	CIFC: 4106	S_H5,6,7,8,9,?	Timor	(*C. arabica* x *C. canephora*)
		HDT hybrid	CIFC: 1343/269	S_H6	Timor	(*C. arabica* x *C. canephora*)
		HDT hybrid	CIFC: 1343/252	S_H?	Timor	(*C. arabica* x *C. canephora*)
		HDT hybrid	CIFC: 19129/4	S_H?	Timor	(*C. arabica* x *C. canephora*)
		HDT hybrid	CIFC: 19138/6	S_H?	Timor	(*C. arabica* x *C. canephora*)
		HDT hybrid	CIFC: 2252/28	S_H?	Timor	(*C. arabica* x *C. canephora*)
		C. arabica Matari	CIFC: 849/1	S_H?	Yemen	
		C. arabica Caturra	CIFC: 19/1	S_H5	Angola	
		C. arabica Bourbon	CIFC: 63/1	S_H5	Brazil	
		C. arabica DK 1/6	CIFC: 32/1	S_H2,5	India	
		C. arabica S288-23	CIFC: 33/1	S_H3,5	India	
		C. arabica S12 Kaffa	CIFC: 134/4	S_H1,4	Ethiopia	
		C. arabica	CIFC: H632/16	S_H3	CIFC	*C. arabica* 849/1 x H536/3 (849/1 x 33/1)
		C. arabica	CIFC: H426/2	S_H5	CIFC	*C. arabica* 19/1 x *C. arabica* 849/1
		C. arabica	CIFC: H426/2-141-11	S_H?	CIFC	F3 of H426/2, susceptible to all rust races
		HDT derivative	CIFC: HW 26/13	S_H5,6,7,9,?	CIFC	*C. arabica* 19/1 x HDT 832/1
		HDT derivative	CIFC: H419/20	S_H5,6,9	CIFC	*C. arabica* Mundo Novo 1535/33 x HDT derivative Hw 26/13
		HDT derivative	CIFC: H420/10	S_H5,6,7,9	CIFC	*C. arabica* Mundo Novo x HDT derivative Hw 26/14
		HDT derivative	CIFC: H537/18	S_H2,5,6	CIFC	*C. arabica* 32/1 x HDT 1343/269
		Cavimor	CIFC: 13727/18	S_H?	Costa Rica	F4 of H528/1 (C. arabica Catuaí S_H5 x HDT derivative Hw 26/13)
		Cavimor	CIFC: 13726/20	S_H?	Costa Rica	F4 of H528/1 (C. arabica Catuaí S_H5 x HDT derivative Hw 26/13)
2	155008	*C. arabica* S353 4/5	CIFC: 34/13	S_H2,3,5	India	
		C. arabica	CIFC: H147/1	S_H2,3,4,5	CIFC	*C. arabica* 34/13 x *C. arabica* 110/5
		HDT derivative	CIFC: H535/10	S_H2,3,5,6	CIFC	*C. arabica* 34/13 x HDT 1343/269
3	155187	*C. arabica* Dilla and Alghe	CIFC: 128/2	S_H1	Kenya	
		HDT derivative	CIFC: H468/41	S_H1,6	CIFC	*C. arabica* 128/2 x HDT 1343/269
4	155124	*C. arabica* S12 Kaffa	CIFC: 635/3	S_H1,4,5	Ethiopia	
		HDT derivative	CIFC: H539/8	S_H1,4,6	CIFC	*C. arabica* 134/4 x HDT 1343/269
5	155134	*C. arabica* S4 Agaro	CIFC: 110/5	S_H4,5	Ethiopia	
		HDT derivative	CIFC: H583/5	S_H4,5,6	CIFC	*C. arabica* 110/5 x HDT 1343/269
6	155188	HDT derivative	CIFC: H420/2	S_H5,8	CIFC	*C. arabica* Mundo Novo 1535/169 x HDT derivative Hw 26/14
7	155133	*C. arabica* Rume Sudan	CIFC: 21336/4	S_H5	Kenya	
8	154942	*C. racemosa*	CIFC: 13969	-	Mozambique	Susceptible to all rust races
9	154952	*C. canephora*	CIFC: 829/1	-	Angola	
10	154959	*C. canephora*	CIFC: 1459	-	Indonesia	
11	154953	*C. canephora*	CIFC: 2975	-	Angola	
12	155123	*C. eugenioides*	CIFC: 16486/23	-	Brazil	
13	155169	*C. eugenioides*	CIFC: 214/43	-	Tanzania	
14	155122	*Coffea* sp.	CIFC: 951/1	-	Dem. Rep. Congo	
15	154815	*C. excelsa*	CIFC: 51/5	-	Madagascar	

Rust resistance factor inheritance: S_H3, from *C. liberica* ancestors; S_H6 to S_H9,from C. *canephora* ancestors; S_H1,S_H2,S_H4, S_H5, from *C. arabica* ancestors; S_H? - verified resistance factor but unknown gene number

Table S1. Chloroplast genome haplotypes for the 61 *Coffea* sp. genotypes from CIFC collection and NCBI GenBank database. Colour code in the haplotype column stands for chloroplast haplotype network shown in Figure 1. Available information on geographic origin, S_H factor and descendants associated with each genotype are provided.

**Haplotype**	**Genome length (bp)**	**Coffee species/variety**	**Genotype Identifier**	S_H factor	**Origin**	**Notes**
16	155011	Kawisari hybrid	CIFC: 644/18	S_H?	Indonesia	(*C. arabica* x *C. liberica*)
17	155189	*C. arabica* CH1	GenBank: MN894550.1	-		
		C. arabica CH2	GenBank: MN894551.1	-		
		C. arabica CM	GenBank: MN894552.1	-		
18	155189	*C. arabica*	GenBank: EF044213.1	-		
		C. arabica	GenBank: NC_008535.1	-		
19	155277	*C. arabica* IN1	GenBank: MK353212.1	-		
20	155192	*C. arabica* JIG2	GenBank: MN370912.1	-		
21	155191	*C. arabica* CH3	GenBank: MK342634.1	-		
22	155191	*C. arabica* HP1	GenBank: MK353209.1	-		
23	155189	*C. arabica* TY1	GenBank: MK875244.1	-		
24	155188	*C. arabica* Bourbon	GenBank: MN901638.1	-		
25	155187	*C. arabica* TY1	GenBank: MK862266.1	-		
26	155186	*C. arabica*	GenBank: MN851270.1	-		
27	154951	*C. racemosa*	GenBank: MK577911.1	-		
28	154942	*C. racemosa*	GenBank: MW970412.1	-		
29	154799	*C. liberica*	GenBank: MW970411.1	-		
30	154751	*C. canephora*	GenBank: KU500324.1	-		
		C. canephora	GenBank: NC_030053.1	-		

Rust resistance factor inheritance: S_H3, from *C. liberica* ancestors; S_H6 to S_H9,from C. *canephora* ancestors; S_H1,S_H2,S_H4, S_H5, from *C. arabica* ancestors; S_H? - verified resistance factor but unknown gene number

Table S2: Chloroplast nuclear-encoded proteins used in this study. For every protein we show an identifier for the protein and the DNA region, chromosome, starting and ending nucleotide, strand (+ or -), *locus* and protein codes, protein length, protein description, number of DNA variants found in the CDS and the up-stream and down-stream flanking regions.

										number of DNA variants		
**Protein_ID**	**DNA_ID**	**Chromosome**	**Starting site**	**Ending site**	**Strand**	**Locus code**	**Protein code**	**Protein Length**	**Description**	Up-stream	CDS	Down-stream
TRX1	DNAregion1	10c	43964886	43968460	+	LOC113714253	XP_027093841.1	200	thioredoxin Y2 chloroplastic-like			
TRX2	DNAregion2	10c	2257338	2258566	-	LOC113713356	XP_027092871.1	221	thioredoxin-like 4 chloroplastic isoform X1			
TRX3	DNAregion2	10c	2257338	2258566	-	LOC113713356	XP_027092872.1	203	thioredoxin-like 4 chloroplastic isoform X2			
IF1	DNAregion3	10c	40015710	40017440	+	LOC113714329	XP_027093931.1	157	translation initiation factor IF-2 chloroplastic-like isoform X2			
IF2	DNAregion3	10c	40015710	40016782	+	LOC113714329	XP_027093928.1	163	translation initiation factor IF-2 chloroplastic-like isoform X1			
IF3	DNAregion3	10c	40015710	40016782	+	LOC113714329	XP_027093929.1	163	translation initiation factor IF-2 chloroplastic-like isoform X1			
IF4	DNAregion3	10c	40015710	40016782	+	LOC113714329	XP_027093930.1	163	translation initiation factor IF-2 chloroplastic-like isoform X1			
TRX4	DNAregion4	10e	3260928	3262155	-	LOC113712756	XP_027092111.1	221	thioredoxin-like 4 chloroplastic isoform X1			
TRX5	DNAregion4	10e	3260928	3262155	-	LOC113712756	XP_027092112.1	203	thioredoxin-like 4 chloroplastic isoform X2			
TRX6	DNAregion5	10e	872800	873705	+	LOC113712447	XP_027091677.1	301	thioredoxin-like protein CDSP32 chloroplastic			
FTSH1	DNAregion6	10e	669428	672622	-	LOC113712758	XP_027092113.1	706	LOW QUALITY PROTEIN: ATP-dependent zinc metalloprotease FTSH, chloroplastic			
TRX7	DNAregion7	11c	14342823	14353061	-	LOC113715333	XP_027095311.1	189	thioredoxin X chloroplastic-like			
NDH1	DNAregion8	11c	28276325	28278266	-	LOC113716766	XP_027097002.1	192	probable NAD(P)H dehydrogenase subunit CRR3, chloroplastic			
IF5	DNAregion9	11c	2149813	2150651	+	LOC113717222	XP_027097737.1	88	translation initiation factor IF-2 chloroplastic-like			
TRX8	DNAregion10	11e	22300176	22309234	+	LOC113717993	XP_027098673.1	189	thioredoxin X chloroplastic-like			
NDH2	DNAregion11	11e	36177716	36181898	-	LOC113719083	XP_027099906.1	173	probable NAD(P)H dehydrogenase subunit CRR3, chloroplastic isoform X2			
NDH3	DNAregion11	11e	36179955	36181898	-	LOC113719083	XP_027099905.1	192	probable NAD(P)H dehydrogenase subunit CRR3, chloroplastic isoform X1			
NDH4	DNAregion11	11e	36180467	36181898	-	LOC113719083	XP_027099907.1	162	probable NAD(P)H dehydrogenase subunit CRR3, chloroplastic isoform X3			
IF6	DNAregion12	11e	38240035	38241271	+	LOC113718601	XP_027099296.1	142	translation initiation factor IF-2 chloroplastic-like			
TRX9	DNAregion13	1c	8583489	8585791	+	LOC113711793	XP_027090835.1	324	thioredoxin-like fold domain-containing protein MRL7L chloroplastic			
NDH5	DNAregion14	1c	45834989	45836590	+	LOC113730405	XP_027110865.1	209	probable NAD(P)H dehydrogenase (quinone) FQR1-like 3 isoform X1			
NDH6	DNAregion14	1c	45834989	45836590	+	LOC113730405	XP_027110874.1	209	probable NAD(P)H dehydrogenase (quinone) FQR1-like 3 isoform X1			
NDH7	DNAregion14	1c	45834989	45836590	+	LOC113730405	XP_027110881.1	209	probable NAD(P)H dehydrogenase (quinone) FQR1-like 3 isoform X1			
NDH8	DNAregion14	1c	45835325	45836590	+	LOC113730405	XP_027110888.1	157	probable NAD(P)H dehydrogenase (quinone) FQR1-like 3 isoform X2			
NDH9	DNAregion14	1c	45835325	45836590	+	LOC113730405	XP_027110896.1	157	probable NAD(P)H dehydrogenase (quinone) FQR1-like 3 isoform X2			
FTSH2	DNAregion15	1c	45734013	45737827	+	LOC113742808	XP_027126600.1	505	ATP-dependent zinc metalloprotease FTSH, chloroplastic-like			
FTSH3	DNAregion16	1c	45723158	45728192	+	LOC113730269	XP_027110657.1	819	ATP-dependent zinc metalloprotease FTSH 9, chloroplastic isoform X1			
NDH10	DNAregion17	1e	42559181	42560758	+	LOC113710959	XP_027089863.1	208	probable NAD(P)H dehydrogenase (quinone) FQR1-like 3 isoform X1			
NDH11	DNAregion17	1e	42559516	42560758	+	LOC113710959	XP_027089869.1	156	probable NAD(P)H dehydrogenase (quinone) FQR1-like 3 isoform X2			
FTSH4	DNAregion18	1e	42464161	42469198	+	LOC113710889	XP_027089756.1	819	ATP-dependent zinc metalloprotease FTSH 9, chloroplastic isoform X1			
FTR1	DNAregion19	2c	24514550	24515086	+	LOC113727096	XP_027106881.1	178	ferredoxin-thioredoxin reductase, variable chain-like			
OEE1	DNAregion20	2c	9618765	9619927	+	LOC113725864	XP_027105067.1	231	oxygen-evolving enhancer protein 3-2, chloroplastic-like			
TRX10	DNAregion21	2c	16302457	16305821	+	LOC113726437	XP_027105954.1	176	thioredoxin M3 chloroplastic-like			
TRX11	DNAregion22	2c	59081288	59081896	+	LOC113727607	XP_027107666.1	202	thioredoxin-like 2-1 chloroplastic			
TRX12	DNAregion23	2c	9860835	9862373	-	LOC113725880	XP_027105085.1	357	thioredoxin-like fold domain-containing protein MRL7 homolog chloroplastic isoform X1	7		
TRX13	DNAregion23	2c	9860835	9862373	-	LOC113725880	XP_027105086.1	357	thioredoxin-like fold domain-containing protein MRL7 homolog chloroplastic isoform X1	7		
TRX14	DNAregion23	2c	9860835	9862373	-	LOC113725880	XP_027105087.1	357	thioredoxin-like fold domain-containing protein MRL7 homolog chloroplastic isoform X1	7		

Table S2: Chloroplast nuclear-encoded proteins used in this study. For every protein we show an identifier for the protein and the DNA region, chromosome, starting and ending nucleotide, strand (+ or -), *locus* and protein codes, protein length, protein description, number of DNA variants found in the CDS and the up-stream and down-stream flanking regions.

										number of DNA variants		
**Protein_ID**	**DNA_ID**	**Chromosome**	**Starting site**	**Ending site**	**Strand**	**Locus code**	**Protein code**	**Protein Length**	**Description**	Up-stream	CDS	Down-stream
TRX15	DNAregion23	2c	9860835	9862373	-	LOC113725880	XP_027105088.1	357	thioredoxin-like fold domain-containing protein MRL7 homolog chloroplastic isoform X1	7		
TRX16	DNAregion23	2c	9861144	9862373	-	LOC113725880	XP_027105089.1	308	thioredoxin-like fold domain-containing protein MRL7 homolog chloroplastic isoform X2	7		
TRX17	DNAregion24	2c	56212459	56213120	+	LOC113724534	XP_027103227.1	141	thioredoxin-like protein CITRX chloroplastic			
NDH12	DNAregion25	2c	9834643	9836415	-	LOC113725878	XP_027105082.1	203	NAD(P)H dehydrogenase (quinone) FQR1-like			
NDH13	DNAregion26	2c	15539836	15542444	+	LOC113726344	XP_027105814.1	275	probable NAD(P)H dehydrogenase (quinone) FQR1-like 2			
FTSH5	DNAregion27	2c	1499387	1508219	-	LOC113724908	XP_027103601.1	839	ATP-dependent zinc metalloprotease FTSH 11, chloroplastic/mitochondrial-like			
FTR2	DNAregion28	2e	26281492	26282028	+	LOC113732269	XP_027113752.1	178	ferredoxin-thioredoxin reductase, variable chain-like			
OEE2	DNAregion29	2e	9587081	9588235	+	LOC113730850	XP_027111606.1	231	oxygen-evolving enhancer protein 3-2, chloroplastic-like			
TRX18	DNAregion30	2e	15940145	15943441	+	LOC113731433	XP_027112513.1	212	thioredoxin M3 chloroplastic-like			
TRX19	DNAregion31	2e	15908024	15911213	+	LOC113731429	XP_027112502.1	176	thioredoxin M3 chloroplastic-like isoform X1			
TRX20	DNAregion31	2e	15910774	15911213	+	LOC113731429	XP_027112503.1	110	thioredoxin M3 chloroplastic-like isoform X2			
TRX21	DNAregion31	2e	15910774	15911213	+	LOC113731429	XP_027112504.1	110	thioredoxin M3 chloroplastic-like isoform X2			
TRX22	DNAregion31	2e	15910635	15911213	+	LOC113731429	XP_027112505.1	108	thioredoxin M3 chloroplastic-like isoform X3			
TRX23	DNAregion31	2e	15910635	15911213	+	LOC113731429	XP_027112506.1	108	thioredoxin M3 chloroplastic-like isoform X3			
TRX24	DNAregion32	2e	8996335	8999657	+	LOC113730788	XP_027111510.1	199	thioredoxin-like 3-2 chloroplastic isoform X1			4
TRX25	DNAregion32	2e	8996335	8999657	+	LOC113730788	XP_027111511.1	198	thioredoxin-like 3-2 chloroplastic isoform X2			4
TRX26	DNAregion33	2e	66654693	66661408	+	LOC113732921	XP_027114768.1	132	thioredoxin-like protein CITRX chloroplastic isoform X1			
TRX27	DNAregion33	2e	66654693	66659830	+	LOC113732921	XP_027114769.1	130	thioredoxin-like protein CITRX chloroplastic isoform X2			
TRX28	DNAregion34	2e	24793647	24795653	+	LOC113732198	XP_027113657.1	132	thioredoxin-like protein Clot			
TRX29	DNAregion35	2e	24834287	24836155	+	LOC113732201	XP_027113659.1	132	thioredoxin-like protein Clot			
NDH14	DNAregion36	2e	9719453	9721225	-	LOC113730860	XP_027111617.1	203	NAD(P)H dehydrogenase (quinone) FQR1-like			
NDH15	DNAregion37	2e	15101330	15103938	+	LOC113731320	XP_027112315.1	275	probable NAD(P)H dehydrogenase (quinone) FQR1-like 2			
FTSH6	DNAregion38	2e	1449532	1458648	-	LOC113729955	XP_027110123.1	805	ATP-dependent zinc metalloprotease FTSH 11, chloroplastic/mitochondrial-like isoform X2			
FTSH7	DNAregion38	2e	1449532	1458648	-	LOC113729955	XP_027110122.1	831	ATP-dependent zinc metalloprotease FTSH 11, chloroplastic/mitochondrial-like isoform X1			
FTSH8	DNAregion38	2e	1451213	1458648	-	LOC113729955	XP_027110124.1	650	ATP-dependent zinc metalloprotease FTSH 11, chloroplastic/mitochondrial-like isoform X3			
TRX30	DNAregion39	3c	41171045	41173596	-	LOC113735229	XP_027118037.1	336	thioredoxin-like fold domain-containing protein MRL7L chloroplastic isoform X1			
TRX31	DNAregion39	3c	41171045	41173596	-	LOC113735229	XP_027118039.1	336	thioredoxin-like fold domain-containing protein MRL7L chloroplastic isoform X1			
TRX32	DNAregion39	3c	41171045	41173184	-	LOC113735229	XP_027118040.1	238	thioredoxin-like fold domain-containing protein MRL7L chloroplastic isoform X2			
IF7	DNAregion40	3c	35621472	35622261	+	LOC113735166	XP_027117938.1	91	translation initiation factor IF-2 chloroplastic-like isoform X2			1
IF8	DNAregion40	3c	35621472	35622261	+	LOC113735166	XP_027117939.1	91	translation initiation factor IF-2 chloroplastic-like isoform X2			1
IF9	DNAregion40	3c	35621472	35622261	+	LOC113735166	XP_027117940.1	91	translation initiation factor IF-2 chloroplastic-like isoform X2			1
IF10	DNAregion41	3e	29197050	29197472	-	LOC113736365	XP_027119100.1	82	translation initiation factor IF-2 chloroplastic-like			
TRX33	DNAregion42	4c	38775415	38776604	+	LOC113740289	XP_027123660.1	275	thioredoxin-like 1-2 chloroplastic			
EFTU1	DNAregion43	4c	8112580	8114058	+	LOC113739865	XP_027123058.1	492	elongation factor TuB			
TRX34	DNAregion44	4e	38843893	38845080	+	LOC113742837	XP_027126639.1	275	thioredoxin-like 1-2 chloroplastic			
TRX35	DNAregion45	4e	1024242	1026581	-	LOC113742864	XP_027126675.1	202	thioredoxin-like 2 chloroplastic			
TRX36	DNAregion46	4e	2158633	2160950	+	LOC113741645	XP_027125032.1	202	thioredoxin-like 2 chloroplastic			
GLYK1	DNAregion47	4e	3335117	3338879	-	LOC113742584	XP_027126253.1	457	D-glycerate 3-kinase, chloroplastic isoform X2	1	5	
GLYK2	DNAregion47	4e	3335117	3338879	-	LOC113742584	XP_027126252.1	485	D-glycerate 3-kinase, chloroplastic isoform X1	1	5	
EFTU2	DNAregion48	4e	10174843	10176321	+	LOC113742241	XP_027125834.1	492	elongation factor TuB			
OEE3	DNAregion49	5c	2721964	2723929	+	LOC113689091	XP_027062723.1	263	oxygen-evolving enhancer protein 2, chloroplastic-like			

Table S2: Chloroplast nuclear-encoded proteins used in this study. For every protein we show an identifier for the protein and the DNA region, chromosome, starting and ending nucleotide, strand (+ or -), *locus* and protein codes, protein length, protein description, number of DNA variants found in the CDS and the up-stream and down-stream flanking regions.

										**number of DNA variants**		
**Protein_ID**	**DNA_ID**	**Chromosome**	**Starting site**	**Ending site**	**Strand**	**Locus code**	**Protein code**	**Protein Length**	**Description**	Up-stream	CDS	Down-stream
FTSH9	DNAregion50	5c	13195160	13205368	+	LOC113690902	XP_027064824.1	1041	ATP-dependent zinc metalloprotease FTSH 12, chloroplastic-like isoform X1			
FTSH10	DNAregion50	5c	13195160	13205368	+	LOC113690902	XP_027064825.1	1015	ATP-dependent zinc metalloprotease FTSH 12, chloroplastic-like isoform X2			
FTSH11	DNAregion51	5c	5657064	5667212	+	LOC113690308	XP_027063944.1	1040	ATP-dependent zinc metalloprotease FTSH 12, chloroplastic-like isoform X1			
FTSH12	DNAregion51	5c	5657064	5667212	+	LOC113690308	XP_027063946.1	1014	ATP-dependent zinc metalloprotease FTSH 12, chloroplastic-like isoform X2			
NDH16	DNAregion52	5e	36781703	36784537	+	LOC113688085	XP_027061537.1	203	probable NAD(P)H dehydrogenase (quinone) FQR1-like 1			
FTSH13	DNAregion53	5c	39640281	39643687	-	LOC113690226	XP_027063860.1	691	ATP-dependent zinc metalloprotease FTSH 2, chloroplastic			
FTSH14	DNAregion54	5e	34391876	34395282	-	LOC113687849	XP_027061170.1	691	ATP-dependent zinc metalloprotease FTSH 2, chloroplastic			
FTSH15	DNAregion55	5e	1707010	1717194	+	LOC113743595	XP_027127447.1	1040	ATP-dependent zinc metalloprotease FTSH 12, chloroplastic-like			
TRR1	DNAregion56	6c	15398202	15407046	+	LOC113691470	XP_027065413.1	571	thioredoxin reductase NTRC-like	1		
TRX37	DNAregion57	6c	7088160	7090853	-	LOC113693942	XP_027068532.1	232	thioredoxin-like 2 chloroplastic			
TRX38	DNAregion58	6c	11873856	11875749	-	LOC113692086	XP_027066239.1	185	thioredoxin-like 3-1 chloroplastic	2		
EFTU3	DNAregion59	6c	13271536	13284857	+	LOC113692806	XP_027067178.1	700	putative elongation factor TypA-like SVR3		1	
EFTU4	DNAregion59	6c	13271536	13284857	+	LOC113692806	XP_027067179.1	690	putative elongation factor TypA-like SVR3		1	
EFTU5	DNAregion59	6c	13272871	13284857	+	LOC113692806	XP_027067180.1	586	putative elongation factor TypA-like SVR3		1	
IF11	DNAregion60	6c	10716249	10721248	-	LOC113694080	XP_027068736.1	269	translation initiation factor IF3-4 chloroplastic-like			
TRR2	DNAregion61	6e	14146831	14155651	+	LOC113694648	XP_027069303.1	572	thioredoxin reductase NTRC-like			
TRX39	DNAregion62	6e	12446935	12448847	-	LOC113695121	XP_027069898.1	185	thioredoxin-like 3-1 chloroplastic			
TRX40	DNAregion63	6e	2807621	2811201	+	LOC113694640	XP_027069287.1	299	thioredoxin-like protein AAED1 chloroplastic isoform X1			
TRX41	DNAregion63	6e	2807621	2811270	+	LOC113694640	XP_027069288.1	291	thioredoxin-like protein AAED1 chloroplastic isoform X2			
TRX42	DNAregion63	6e	2807621	2811176	+	LOC113694640	XP_027069289.1	260	thioredoxin-like protein AAED1 chloroplastic isoform X3			
EFTU6	DNAregion64	6e	15662169	15675583	-	LOC113695988	XP_027071061.1	690	putative elongation factor TypA-like SVR3			
EFTU7	DNAregion64	6e	15662169	15675583	-	LOC113695988	XP_027071060.1	700	putative elongation factor TypA-like SVR3			
IF12	DNAregion65	6e	11227040	11231996	-	LOC113697307	XP_027072682.1	269	translation initiation factor IF3-4 chloroplastic-like			
FTR3	DNAregion66	7c	610043	612670	-	LOC113698720	XP_027074434.1	149	ferredoxin-thioredoxin reductase catalytic chain, chloroplastic-like			
TRX43	DNAregion67	7c	4667704	4670160	+	LOC113699117	XP_027075035.1	291	thioredoxin-like protein AAED1 chloroplastic			
TRX44	DNAregion68	7c	17803768	17807346	-	LOC113700207	XP_027076525.1	274	thioredoxin-like protein HCF164 chloroplastic	61	30	23
TRR3	DNAregion69	7c	8626330	8628369	-	LOC113699342	XP_027075440.1	367	thioredoxin reductase NTRB-like			
NDH17	DNAregion70	7c	6271254	6273873	-	LOC113700106	XP_027076368.1	252	probable NAD(P)H dehydrogenase (quinone) FQR1-like 2			
FTSH16	DNAregion71	7c	4492540	4493379	+	LOC113698940	XP_027074720.1	279	ATP-dependent zinc metalloprotease FTSH, chloroplastic-like			
FTSH17	DNAregion72	7c	8846188	8848695	-	LOC113698659	XP_027074349.1	681	ATP-dependent zinc metalloprotease FTSH 6, chloroplastic-like			
OEE4	DNAregion73	7e	1086466	1088420	-	LOC113702187	XP_027079053.1	331	oxygen-evolving enhancer protein 1, chloroplastic			
TRX45	DNAregion74	7e	2823186	2825552	+	LOC113701817	XP_027078439.1	262	thioredoxin-like protein AAED1 chloroplastic			
TRX46	DNAregion75	7e	15167249	15170826	+	LOC113700346	XP_027076573.1	274	thioredoxin-like protein HCF164 chloroplastic			3
TRR4	DNAregion76	7e	5838755	5840795	-	LOC113702165	XP_027079000.1	367	thioredoxin reductase NTRB-like			
FTSH18	DNAregion77	7e	6033086	6035616	-	LOC113701458	XP_027077924.1	681	ATP-dependent zinc metalloprotease FTSH 6, chloroplastic-like			
TRX47	DNAregion78	8c	34840006	34842934	+	LOC113705500	XP_027083165.1	184	thioredoxin-like protein CITRX chloroplastic isoform X1			
TRX48	DNAregion78	8c	34840006	34842934	+	LOC113705500	XP_027083166.1	143	thioredoxin-like protein CITRX chloroplastic isoform X2			
EFTU8	DNAregion79	8c	36168756	36172766	-	LOC113706771	XP_027084560.1	791	elongation factor G-2			
IF13	DNAregion80	8c	27507616	27508861	-	LOC113706602	XP_027084335.1	146	translation initiation factor IF-2 chloroplastic-like			
NDH18	DNAregion81	8e	41228084	41229572	+	LOC113702699	XP_027079628.1	203	NAD(P)H dehydrogenase (quinone) FQR1-like			
EFTU9	DNAregion82	8e	41699908	41703910	-	LOC113703655	XP_027080895.1	790	elongation factor G-2			
EFTU10	DNAregion82	8e	41699908	41703910	-	LOC113703655	XP_027080894.1	791	elongation factor G-2			
TRX49	DNAregion83	9c	3560132	3562810	+	LOC113708787	XP_027087186.1	175	thioredoxin F chloroplastic-like			

Table S2: Chloroplast nuclear-encoded proteins used in this study. For every protein we show an identifier for the protein and the DNA region, chromosome, starting and ending nucleotide, strand (+ or -), *locus* and protein codes, protein length, protein description, number of DNA variants found in the CDS and the up-stream and down-stream flanking regions.

										number of DNA variants		
**Protein_ID**	**DNA_ID**	**Chromosome**	**Starting site**	**Ending site**	**Strand**	**Locus code**	**Protein code**	**Protein Length**	**Description**	Up-stream	CDS	Down-stream
TRX50	DNAregion84	9c	37837915	37839664	-	LOC113708909	XP_027087423.1	322	thioredoxin-like 1-1 chloroplastic			
TRX51	DNAregion86	2e	9743092	9744627	-	LOC113730862	XP_027111619.1	357	thioredoxin-like fold domain-containing protein MRL7 homolog chloroplastic			
TRX52	DNAregion86	2e	9743092	9744627	-	LOC113730862	XP_027111620.1	357	thioredoxin-like fold domain-containing protein MRL7 homolog chloroplastic			
TRX53	DNAregion86	2e	9743092	9744627	-	LOC113730862	XP_027111621.1	357	thioredoxin-like fold domain-containing protein MRL7 homolog chloroplastic			
TRX54	DNAregion86	2e	9743092	9744627	-	LOC113730862	XP_027111622.1	357	thioredoxin-like fold domain-containing protein MRL7 homolog chloroplastic			
TRX55	DNAregion86	2e	9743092	9744627	-	LOC113730862	XP_027111623.1	357	thioredoxin-like fold domain-containing protein MRL7 homolog chloroplastic			
TRX56	DNAregion87	9e	35526253	35527947	-	LOC113709536	XP_027088124.1	321	thioredoxin-like 1-1 chloroplastic			
TRX57	DNAregion88	9e	11436454	11444467	-	LOC113709657	XP_027088278.1	130	thioredoxin-like protein CITRX chloroplastic isoform X1			
TRX58	DNAregion88	9e	11443698	11444467	-	LOC113709657	XP_027088279.1	130	thioredoxin-like protein CITRX chloroplastic isoform X2			
FTR4	DNAregion90	NW_020850478.1	1828684	1831306	-	LOC113722812	XP_027101834.1	155	ferredoxin-thioredoxin reductase catalytic chain, chloroplastic-like			
ISP	DNAregion91	5e	31773064	31776765	-	LOC113743416	XP_027127212.1	232	cytochrome b6-f complex iron-sulfur subunit 1, chloroplastic			

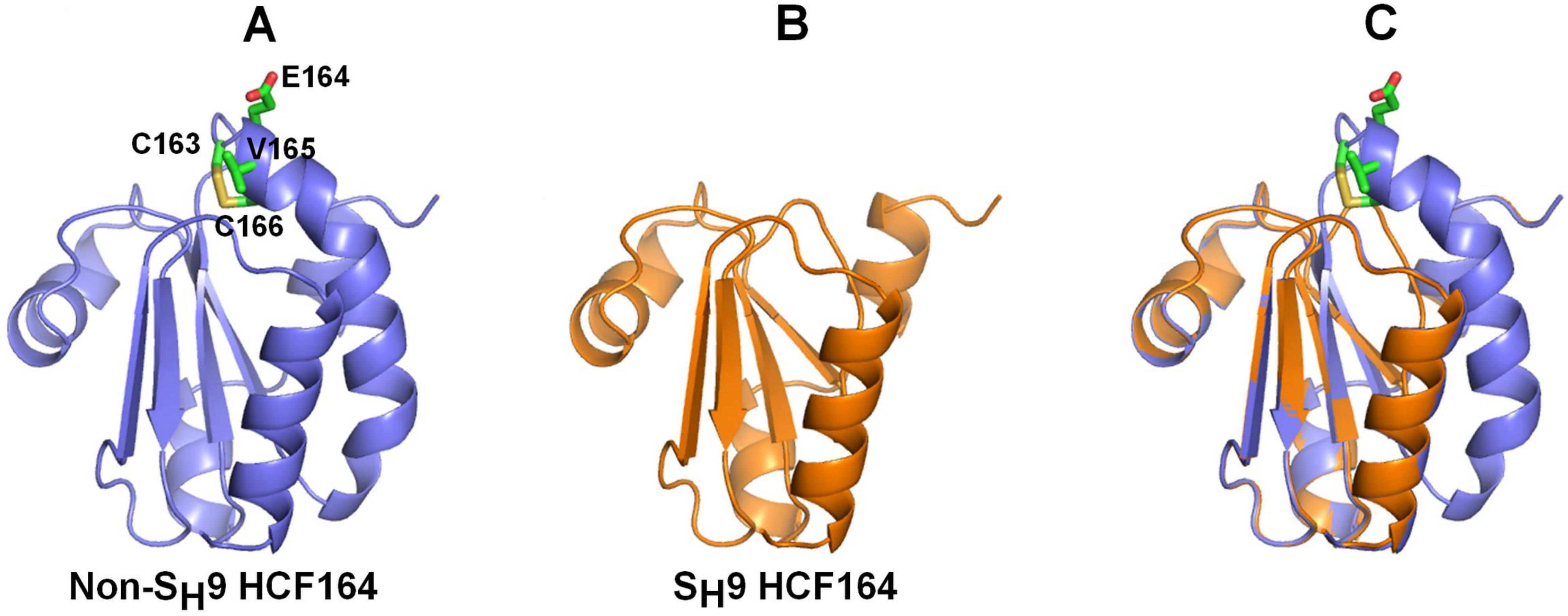

Figure S1: Models for HCF164 protein in non-SH9 individuals (A) and SH9 individuals (B). The CEVC residues forming the active center of the HCF164 protein in the non-SH9 individuals are highlighted with sticks. **C**) Superposition of the HCF164 protein models of non-SH9 and SH9 individuals. Note that in the HCF164 model for SH9 individuals, the α-helix containing the active center is not present due to the 19-residue deletion identified in this work.
